# Hypoxia activates placental growth factor expression in lymphatic endothelial cells

**DOI:** 10.18632/oncotarget.15861

**Published:** 2017-03-02

**Authors:** Laura Tudisco, Augusto Orlandi, Valeria Tarallo, Sandro De Falco

**Affiliations:** ^1^ Angiogenesis LAB, Institute of Genetics and Biophysics ‘Adriano Buzzati-Traverso’, CNR, Naples, Italy; ^2^ Department of Biomedicine and Prevention, Anatomic Pathology, University of Tor Vergata, Rome, Italy

**Keywords:** hypoxia, PlGF, VEGF family, lymphatic cells, colorectal cancer

## Abstract

Placental growth factor (PlGF), a proangiogenic member of vascular endothelial growth family, is active during pathological conditions like cancer, metastasis formation and hind limb ischemia and in wound healing. Endothelial cells express PlGF and hypoxia positively modulates *in vitro* its expression. To verify whether hypoxia modulates PlGF expression in different cellular contexts and *in vivo*, we first analyzed five human and five mouse cancer cell lines showing that in eight of them hypoxia positively modulates PlGF. Next, we analyzed xenograft colorectal cancer tumors showing that human cancer cells were able to express PlGF in hypoxic area of the tumor. Surprisingly, we did not visualize mouse PlGF in CD31 positive tumor vessels, but in low CD31 positive vessels, a characteristic of lymphatic vessels. We found that hypoxia effectively activates PlGF expression in lymphatic endothelial cells as well as in LYVE1 positive tumor vessels. We also investigated two additional mouse angiogenic models, hind limb ischemia and wound healing, and we confirmed that lymphatic vessels of both ischemic muscles and skin express PlGF. These results show for the first time that hypoxia activates PlGF expression in lymphatic endothelial cells, which have to be considered an additional source for PlGF production in pathological contexts.

## INTRODUCTION

Placental growth factor (PlGF) is a member of vascular endothelial growth factor (VEGF) family. It is a specific ligand for the common receptor of pro-angiogenic members (VEGF-A, VEGF-B and PlGF) of the family, the high affinity VEGF receptor, VEGFR1. Its activity is synergic with that of VEGF-A, the main member of VEGF family, which is also able to bind and activate the low affinity VEGF receptor, VEGFR2, the main receptor driving neo-vessels formation from pre-existing ones [1].

PlGF is redundant for physiological processes [2] but has an active role confined to pathological conditions [3]. Genetic ablation or biochemical inhibition of PlGF impair angiogenesis and arteriogenesis associated to pathological conditions such as tumor growth, heart, limb and ocular ischemia [3–7]. Delivery of recombinant PlGF or adenovirus-mediated PlGF delivery elicit a strong angiogenic response in ischemic conditions and in cancer similar to that of VEGF-A [4, 8–10]. In tumor context, a strict relationship between PlGF/VEGFR1 axis and metastasis formation has also been reported. VEGFR1 activation and PlGF markedly promotes pulmonary metastases through induction of matrix metalloproteinase-9 secretion [11, 12] and plays a crucial role in the establishment of pre-metastatic niches [13]. The functional role of PlGF/VEGFR1 in tumor metastasis establishment was further confirmed using different kind of inhibitors [14–18].

Hypoxia is one of the key drivers of neo-angiogenesis in pathological conditions. This condition, through the activation of different mediators, induces the accumulation of hypoxia inducible factor (HIF) transcriptional activators in the cells surrounding the hypoxic area, which determine the overexpression of a large number of genes [19, 20]. Despite is known that a strict biochemical and functional relationship between VEGF-A and PlGF exists and that hypoxia strongly upregulates *Vegf-a* and *Vegfr1* expression [21, 22], only recently we have been able to define the molecular basis governing the modulation of *Plgf* expression exerted by hypoxia in human and mouse endothelial cells. The hypoxic *Plgf* overexpression is specifically driven by HIF-1α, which is able to recognize a hypoxia responsive element (HRE) located in the second intron of PlGF gene [23].

Regarding the relationship between hypoxia and PlGF expression *in vivo*, few and non conclusive data have been produced until now. Indeed, in the few reports in which the visualization of PlGF by immunohistochemical (IHC) or immunofluorescence (IF) analyses was performed, no correlation was made with the hypoxic status of analyzed tissues [24–27]. In addition, to our knowledge, no one has been able to visualize PlGF expression into the vessels of normoxic or hypoxic tissues, despite it is well-established that endothelial cells express PlGF *in vitro* and that it is upregulated in hypoxic condition [23, 28, 29].

Therefore, we decided to verify whether hypoxia induces expression of PlGF in other cellular contexts and, most importantly, *in vivo*. Initially, we focused our attention on tumor context analyzing several human and mouse tumor cell lines. We then analyzed xenograft colorectal cancer by IHC and IF analyses to evaluate the expression of both human and mouse PlGF. The observation that mouse PlGF staining was exclusively associated with low CD31 positive staining, a characteristic of lymphatic endothelial cells (LECs), allowed us to investigate whether hypoxia could activate the expression of PlGF in LECs *in vitro*, and in lymphatic vessels *in vivo*, by analyzing two pathological angiogenic models, tumor growth and hind limb ischemia and wound healing [30].

## RESULTS

### Hypoxia induces PlGF expression in several cancer cell lines and *in vivo* in colorectal cancer cells

**Figure 1:**
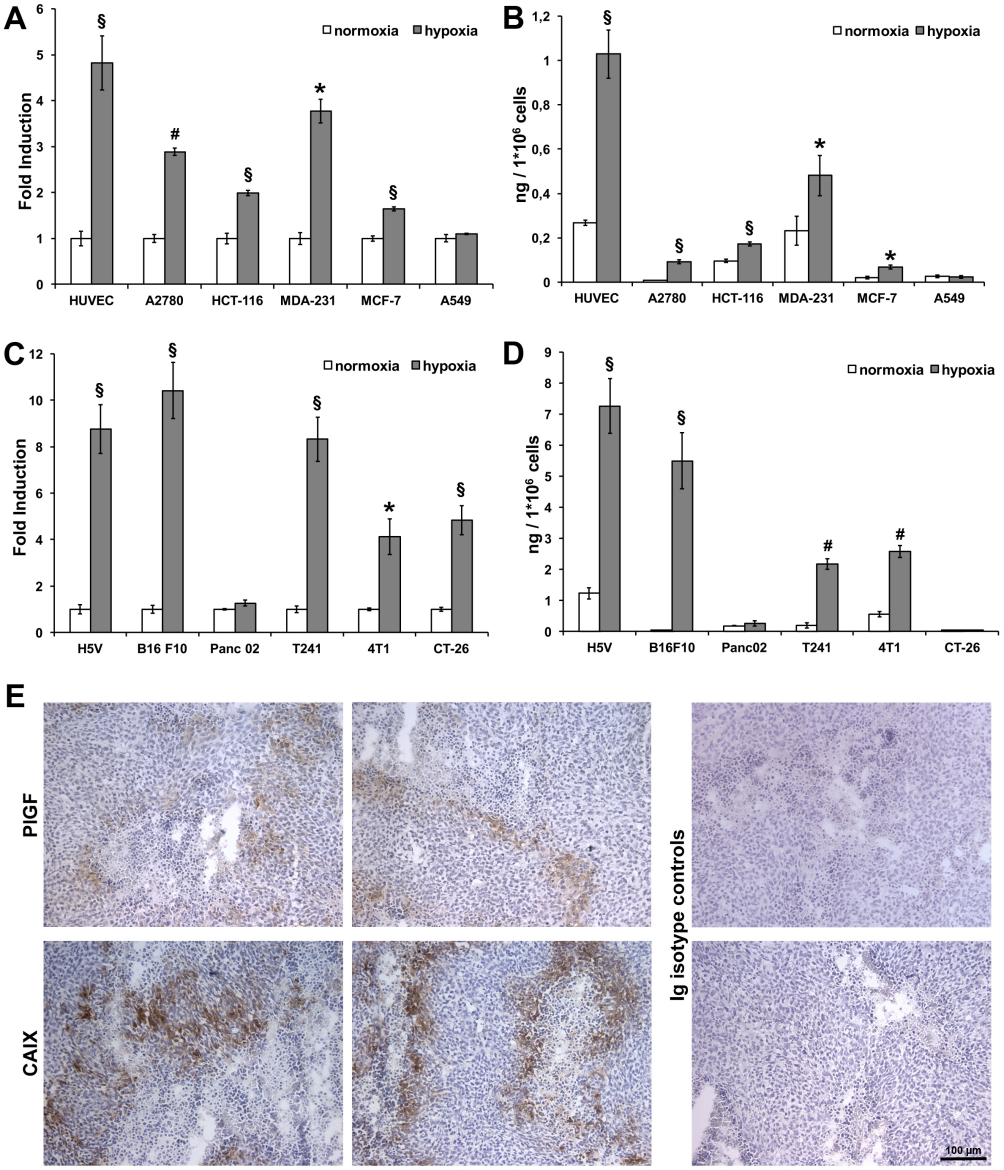
Hypoxia upregulates PlGF expression in human and mouse cancer cell lines and in xenograft colorectal cancer RT-qPCR was performed on RNA extracted from human **(A)** and mouse **(C)** endothelial and cancer cell lines cultured in normoxic (white bars) and hypoxic (gray bars) (1% O_2_) condition for 12 hours. Data are expressed as fold induction compared with normoxic condition and normalized against RPL32, in human cells, and Rpl13a, in mouse cells. Values represent the mean ± SEM of two independent experiments performed in triplicate. *p<0.05, ^§^p<0.005 and ^#^p<0.0005 compared to normoxia condition. Sandwich ELISAs for quantification of hPlGF and mPlGF were performed on culture media of human **(B)** and mouse **(D)** endothelial and cancer cell lines cultured in normoxic (white bars) and hypoxic (gray bars)(1% O_2_) condition for 24 hours. Values represent the mean ± SEM of two independent experiments performed in duplicate. *p<0.05, ^§^p<0.005 and ^#^p<0.0005 compared to normoxia condition. **(E)** Representative images of immunohistochemical analyses for hPlGF and CAIX staining performed on cryosections of xenograft colorectal cancer. Scale bar 100μm.

Human and mouse cancer cell lines of different origin were cultured in normoxic and hypoxic (1% O_2_) conditions and the expression of PlGF was evaluated by RT-qPCR and ELISA (Figure 1). Human cell lines derived from ovaric (A2780), colorectal (HCT-116), breast (MDA231 and MCF7) and lung (A549) cancers and mouse cell lines derived from melanoma (B16F10), fibrosarcoma (T241) and pancreatic (PancO2), breast (4T1) and colorectal (CT-26) cancers were assayed. As positive control, HUVEC and mouse H5V endothelial cells were used. We performed RT-qPCR on RNA extracted after 12 hours, and ELISA on culture media after 24 hours of exposure to hypoxia, based on our previous report [23].

Human PlGF (hPlGF) was significantly upregulated in hypoxic conditions at RNA and protein level in all cell lines except for A549 lung cancer cells (Figure 1A-1B). Instead, mouse PlGF (mPlGF) was significantly upregulated in all cells assayed, except for the pancreatic cell line. In CT-26 cells, the upregulation in hypoxic conditions was observed only at RNA level, whereas at protein level PlGF was not detected, probably because the quantity of protein produced by these cells was under the detection limit of the ELISA (Figure 1C-1D). These data clearly show that PlGF is effectively upregulated by hypoxia *in vitro* in almost all the human and mouse tumor cell lines assayed, confirming what we previously observed in endothelial cells [23].

To verify whether *in vivo* tumor cells undergoing hypoxic conditions express PlGF, we performed analyses of xenograft colorectal tumors generated by subcutaneous injection of HCT-116 cells. This tumor model was chosen for its importance in anti-angiogenic therapeutic approaches. This analysis was performed using an antibody specific for human PlGF exclusively by immunohistochemistry (IHC) analysis, because we did not find antibodies working in immunofluorescence (IF) protocols.

As shown in Figure 1E, we were able to clearly detect hPlGF expression limited to tumor areas in which low concentration of oxygen is expected to be present close to the necrotic areas. Indeed, the labeling was similar to that obtained by immunodetection of carbonic anhydrase IX (CAIX) [31], a hypoxia-inducible enzyme overexpressed by many cancer cells types, which clearly delimited the hypoxic area that surrounded the necrotic areas of the tumor. The isotype-matched antibodies for anti-PlGF and the anti-CAIX antibodies did not show any stain.

These data demonstrate that PlGF is effectively expressed *in vivo*, at least in human colorectal cancer cells, and that its expression is restricted to the areas of the tumors where the highest levels of hypoxia occur.

### PlGF co-localizes with low CD31 positive vessels

**Figure 2:**
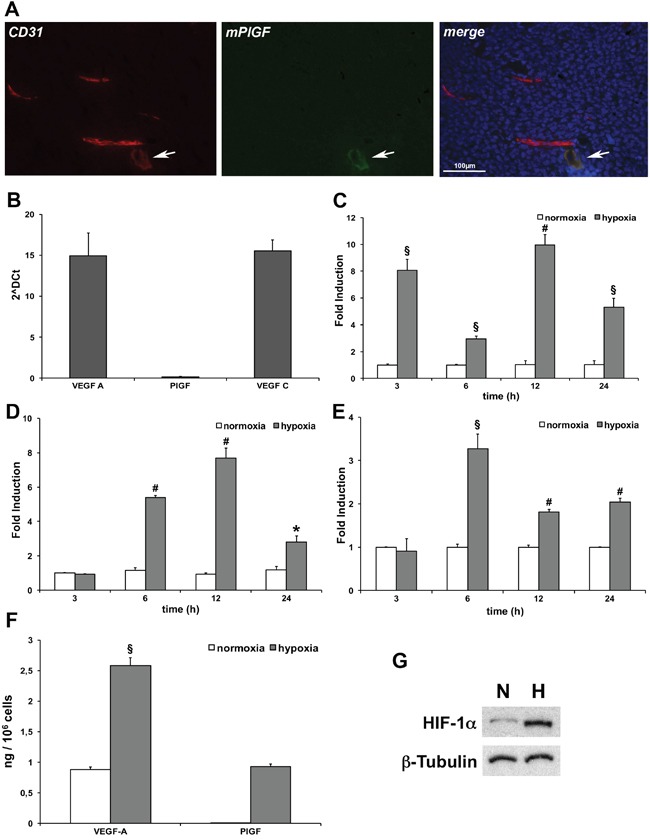
PlGF is expressed in low CD31 positive tumor vessels and hypoxia activates its expression in LECs **(A)** Co-localization of PlGF (green) and CD31(red) on cryosections of xenograft colorectal cancer assessed by immunofluorescence analysis. Scale bar 100μm. White arrows indicate small vessel expressing both the markers. **(B)** Evaluation of mouse VEGF-A, VEGF-C and PlGF expression levels of LECs in normoxic condition assessed by RT-qPCR. Values represent the mean ± SEM of two independent experiments performed in triplicate. Time-dependent effect of hypoxia (gray bars) (1% O_2_) on VEGF-A **(C)** PlGF **(D)** and VEGF-C **(E)** mRNA expression compared to normoxic expression (white bars) assessed by RT-qPCR. Data are expressed as fold induction and normalized to Rpl13a in mouse cells. Values represent the mean ± SEM of two independent experiments performed in triplicate. *p<0.05, ^§^p<0.005 and ^#^p<0.0005 compared to normoxia condition. **(F)** Sandwich ELISA for quantification of mouse VEGF-A and PlGF performed on culture media of LECs cultured in normoxic (white bars) and hypoxic (gray bars)(1% O_2_) condition for 24 hours. Values represent the mean ± SEM of two independent experiments performed in duplicate. ^§^p<0.005 vs normoxia condition. **(G)** Western blot analysis of mouse HIF-1α performed on LECs protein extracts after cells exposure to 1% O_2_ for 24 hours. β-Tubulin was used as loading control.

In order to investigate whether mPlGF resulted expressed in mouse endothelial cells, hence in blood tumor vessels, HCT-116 xenograft tumor cryosections were stained with an antibody specific for mPlGF. In this case, we were able to perform immunofluorescence analyses that allowed us to carry out co-localization experiments with the pan-endothelial marker CD31. Differently from what was observed *in vitro*, PlGF never co-localized with CD31 positive vessels (Figure 2A), despite the several tumor sections analyzed. Surprisingly, we were able to detect PlGF expression only in vessels showing low CD31 staining, a characteristic of lymphatic vessels (Figure 2A). Therefore, we suspected that hypoxia could activate PlGF expression in LECs.

### Hypoxia activates PlGF expression in cultured lymphatic endothelial cells

The expression of PlGF in cultured mouse LECs was analyzed. As expected, in normoxic conditions the expression of mRNA for VEGF-A and VEGF-C was detected by RT-qPCR, while the expression of PlGF was undetectable, confirming previous detailed molecular characterization of LECs [32] (Figure 2B). Next, we performed a time-dependent experiment in hypoxic condition (1% O_2_) evaluating the mRNA expression for VEGF-A, PlGF and VEGF-C (Figure 2C, 2D and 2E, respectively). As expected, both VEGF-A and VEGF-C were significantly upregulated by hypoxia [21, 33] in LECs. VEGF-A was strongly upregulated already after 3 hours, and showed a second peak of expression after 12 hours of hypoxia. VEGF-C started to be expressed between 3 and 6 hours of exposure to low oxygen tension, showing the peak of upregulation after 6 hours. Its expression remained significantly upregulated up to 24 hours of hypoxia. Surprisingly, we found that PlGF started to be expressed between 3 and 6 hours after LECs exposure to hypoxia, and that the expression was significantly upregulated up to 24 hours. As a consequence of PlGF mRNA upregulation we were able to detect the presence of PlGF protein in the culture medium after 24 hours of hypoxia exposure. As expected, the levels of VEGF-A were found increased (Figure 2F). The LECs hypoxic condition was confirmed by western blot analysis for HIF-1α (Figure 2G).

### PlGF is expressed in tumor LYVE1 positive vessels

**Figure 3:**
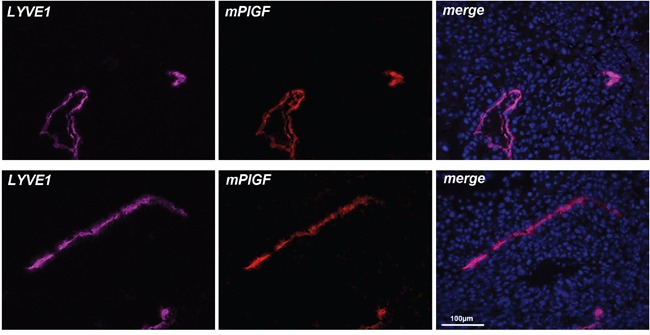
PlGF is expressed in LYVE1 positive tumor lymphatic vessels LYVE1 (pink) staining identify lymphatic vessels on cryosections of xenograft colorectal cancer. PlGF (red) fully co-localizes with LYVE1. Scale bar 100μm.

Therefore, we decided to evaluate if PlGF expression is detectable *in vivo* in lymphatic vessels that were visualized using an anti-LYVE1 antibody. First, we analyzed xenograft colorectal tumor tissues, taking into account the high level of hypoxia normally present in tumor microenvironment. As shown in Figure 3, LYVE1 staining clearly identified lymphatic vessels and PlGF staining perfectly co-localized to that of LYVE1.

### PlGF is expressed in lymphatic vessels also in hind limb ischemia and in wound healing models

To confirm the data observed in cells and tumor tissue, we decided to investigate whether mPlGF is expressed in lymphatic vessels in an additional angiogenic pathological mouse models, the hind limb ischemia, and in wound healing.

**Figure 4:**
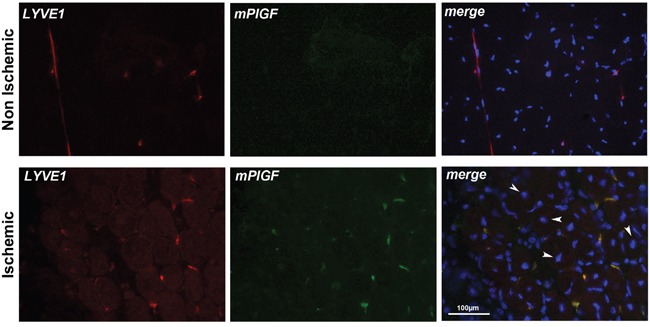
PlGF co-localizes with LYVE1 positive lymphatic vessels in ischemic muscle LYVE1 (red) identifies small inter-fiber lymphatic vessels in non-ischemic and ischemic popliteal muscles cryosections. PlGF (green) is absent in non-ischemic muscle while co-localizes with LYVE1 staining in ischemic muscle. Arrows indicate center-nucleated fibers, a characteristic of regenerating muscle that occurs in hypoxic area of muscles, that were present only in ischemic muscles. Scale bar 100μm.

For hind limb ischemia model, we analyzed popliteal muscle sections from mouse legs that underwent femoral artery ligation. As control, non-ischemic popliteal muscle from contralateral legs that underwent sham surgery were analyzed. Muscles were explanted after seven days from femoral artery ligation. First of all, we performed a co-localization experiment to visualize CD31 and mPlGF and, as occurred for tumor sections, we were not able to detect CD31 positive vessels expressing mPlGF (data not shown). In non-ischemic muscles, LYVE1 was detected in inter-fibers lymphatic vessels whereas mPlGF was absent. Conversely, in ischemic muscles, PlGF was detectable and fully co-localized with LYVE1 positive vessels (Figure 4). Interestingly, only in the ischemic muscle sections center-nucleated fibers are observable, a classical aspect of regenerating muscle that normally occurs in hypoxic area of muscles undergoing ischemic conditions [34].

**Figure 5:**
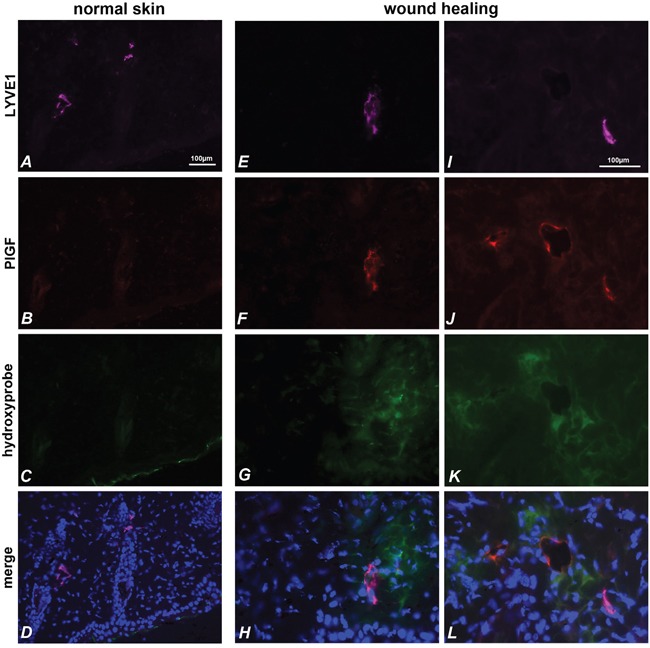
PlGF and LYVE1 co-localize in hypoxic area of skin during wound healing LYVE1 (pink) **(A, E, I)** identifies lymphatic vessels in normal skin **(A-D)** and in wound healing skin **(E-L)**. PlGF (red) is absent in normal skin **(B)**, but is expressed in wound healing skin **(F, J)** and co-localizes with LYVE1 staining **(D, H, L)**. The stained vessels result surrounded by hypoxic areas identified with hypoxyprobe **(C, G, K)**. PlGF also stains vessels in hypoxia area of skin that are negative for LYVE1 marker **(J, L)**.

Finally, we analyzed mouse skin sections in wound healing. The analysis was performed on skin explanted two days after skin incision, and as control on normal skin. Hypoxic areas were identified using hypoxyprobe, thanks to intraperitoneal injection of pimonidazole. As shown in Figure 5A-5D, in normal skin mPlGF was not detected in LYVE1 positive vessels. Conversely, in wound healing mPlGF was expressed in LYVE1 positive vessels surrounded by hypoxic area (Figure 5E-5L). Interestingly, as shown in Figure 5J-5L, anti-mPlGF clearly identified three vessels in a hypoxic area marked by hypoxyprobe, but only one is positive for LYVE1 marker. This indicates that, at least in skin, hypoxia is probably able to drive PlGF expression in blood vessels, too.

## DISCUSSION

Despite the several reports stating that the expression of PlGF is upregulated *in vitro* under hypoxic condition in endothelial cells [28, 29], fibroblasts [35], cardiomyocytes [27] and in cancer cell lines [36, 37], only occasional data are available on visualization of PlGF *in vivo* in tissues in which pathological angiogenesis or wound healing, and consequently hypoxia, occurs. This was due to absence of efficient anti-PlGF antibodies for IHC analyses. To overcome this limit, we have evaluated the reactivity of several commercially available anti-human and anti-mouse PlGF antibodies and we established conditions that allowed to detect human and mouse PlGF by IHC or IF staining, respectively.

Here, we report that in eight out of ten human and mouse tumor cell lines analyzed *in vitro*, hypoxia significantly upregulated PlGF expression, confirming the trend previously observed in cultured human and mouse endothelial cells. About human tumors, IHC analyses have previously evidenced the presence of PlGF in hepatocellular carcinoma [24], advanced stage of human colorectal carcinomas [25] and in pediatric medulloblastoma [26], but its expression has never been associated to hypoxia status of the tumors. We found that the expression of PlGF in xenograft colorectal carcinoma was detectable exclusively in tumor area in which low concentration of oxygen is present. Indeed, PlGF staining was similar to that of CAIX, a well-established marker of hypoxia, confirming the link among hypoxia and PlGF expression in cancer cells *in vivo*, at least in this cancer model.

Based on our previous data on endothelial cells *in vitro* [23], we expected to be able to visualize mouse PlGF in tumor blood vessels but we never detected PlGF expression in CD31 positive vessels. This also occurred in ischemic muscle after hind limb ischemia. Surprisingly, we observed expression of PlGF in low CD31 positive vessels, namely in lymphatic vessels, as confirmed by co-localization with LYVE1 marker. These results were corroborated by *in vitro* data that clearly showed how hypoxia activates PlGF expression in LECs. The expression of PlGF in lymphatic vessels under hypoxic conditions occurred also in hind limb ischemia, indicating that this represents a general mechanism of PlGF activation in neo-vascular pathological context, and in wound healing model.

The evidence of PlGF expression in tumor lymphatic vessels represents an interesting achievement considering the various data that correlate the function of PlGF and that of its specific receptor VEGFR1 in metastatization process. Indeed, the active role of PlGF for the recruitment and maturation of bone marrow derived progenitors involved in angiogenic and metastatic processes has been demonstrated [38, 39]. The specific inhibition of VEGFR-1 achieved with different classes of inhibitors strongly impairs tumor cell transmigration from blood compartment to target organs and metastasis establishment and growth [14, 16–18]. In addition, several reports indicate that in patients the expression of PlGF, or its specific inhibition, correlate with the metastatization process associated to different tumors as larynx carcinoma [40], squamous cell carcinoma [41], thyroid carcinoma [42], ovarian cancer [43] and non-small cell lung cancer [12]. Since lymphatic vessels play a central role in metastasis spread, the observation that the cells composing them start to express PlGF under hypoxic conditions *in vivo*, further reinforces the role of PlGF/VEGFR1 axis in metastatization process emphasizing the importance of therapeutic PlGF inhibition.

Finally, only in skin specimens we observed vessels stained by anti-PlGF antibody that are negative for LYVE1 marker, probably blood vessels. This observation highlights an additional interesting point on how the different microenvironment of tissues may influence the expression of specific genes and this will be further investigated in the future to unveil the molecular mechanisms governing this phenomenon.

## MATERIALS AND METHODS

### Cell culture

All tumor cells were purchased from ATCC. Human umbilical vein endothelial cells (HUVECs, Lonza) were cultured in endothelial basal medium (EBM-2) supplemented with endothelial growth factors (EGM-2 bullet kit, Lonza). HUVECs at passages 4–7 were used for all the experiments. Murine-immortalized heart microvascular endothelial cells (H5V) [44] and lymphatic endothelial cells (LECs) [45], murine breast cancer (4T1), human breast cancer (MDA231 and MCF-7) and adenocarcinoma human alveolar basal epithelial (A549) cell lines were cultured in DMEM, supplemented with 10% heat-inactivated fetal bovine serum, 2 mM L-glutamine and standard concentration of antibiotics. Human ovaric cancer cell line (A2780), murine melanoma (B16F10), pancreatic (Panc02), colorectal (CT-26) and fibrosarcoma (T241) tumor cell lines were cultured in RPMI, supplemented with 10% heat-inactivated fetal bovine serum, 2 mM L-glutamine and standard concentration of antibiotics this information is reported below. HCT-116 (human colorectal carcinoma) was cultured in McCoy's supplemented with 10% heat-inactivated fetal bovine serum, 2 mM L-glutamine and standard concentration of antibiotics. All reagents for cell culture except those for endothelial and lymphatic cell lines, were from Euroclone. For exposure to hypoxia, sub-confluent cells were placed in an appropriate incubator at 1% oxygen concentration. As control, sub-confluent cells were cultured in normoxic condition.

### RNA preparation and reverse transcription quantitative real-time PCR (RT-qPCR)

Total RNA was isolated using Trizol reagent (Invitrogen), DNase treated and reverse transcribed. The RT products (cDNA) were amplified on CFX96TM Real Time PCR Detection Systems (BioRad) with SYBR green Master Mix (BioRad) using the following oligonucleotide primers specific for: human PlGF (forward 5′-ATGTTCAGCCCATCCTGTGT-3′ and reverse 5′-CTTCATCTTCTCCCGCAGAG-3′), human VEGF-A (forward 5′-AGGGCAGAATCATCACGAAG-3′ and reverse 5′-ATCCGCATAA TCTGCATGGT-3′), human RPL-32 (forward 5′-AGTTCCTGGTCCACAACGTC-3′ and reverse 5′-TGCACATGAGCTGCCTACTC-3′, mouse PlGF (forward 5′-GCTGGTCATGAAGCTGTTC-3′ and reverse 5′-ACCCCACACTTCGTTGAAAG-3′) mouse VEGF-A (forward 5′-CAGGCTGCTGTAACGATGAA-3′ and reverse 5′-GCATTCACATCTGCTGTGCT-3′), mouse VEGF-C (forward 5′-TGCCGGTGCATGTCTAAACT-3′ and reverse 5′-CAGGCATCGGCACATGTAGT-3′) mouse Rpl13a (forward 5′-CCCTCCACCCTATGACAAGA-3′ and reverse 5′-CTGCCTGTTTCCGTAACCTC-3′). The qPCR cycling conditions were 50°C for 2 minutes, 95°C for 10 minutes followed by 40 cycles of a two-step amplification program (95°C for 15 s and 58°C for 1 minute). At the end of the amplification, melting curve analysis was applied using the dissociation protocol from the sequence detection system to exclude contamination with unspecific PCR products. The PCR products were also confirmed by agarose gel and showed only one specific band of the predicted size. For negative controls, no RT products were used as templates in the qPCR and verified by the absence of gel-detected bands. Relative expressions of target genes were determined by the 2–ΔΔCt method. The expression levels in hypoxic condition were calculated with respect to the normoxic level and normalized against RPL32, in human cells, and Rpl13a, in mouse cells. Each point was done in triplicate.

### ELISA assay

All the reagents used in ELISA were from R&D Systems. The assays were performed as described elsewhere [9, 46].

### Animals

C57Bl6/J and CD1 nude athymic mice were purchased from Charles River. Animal experiments were in accordance with European directives no. 2010/63/UE and with Italian directives D.L. 26/2014. For all procedures, anesthesia was performed by intraperitoneal injection of 100 mg/kg ketamine hydrochloride and 10mg/kg xylazine.

### Xenograft tumor model

7- to 8-week-old CD1 nude athymic mice (N=3) were injected subcutaneously with 4 × 10^6^ HCT-116 cells into the right flank. Tumor growth was monitored quantifying tumor volume (mm^3^) three times a week by measuring tumor shortest (d) and longest (D) diameters with an electronic caliper, using the formula D x d^2^/2 (see also [18]). When tumor reached a volume of about 1000 mm^3^ were explanted and processed for immunohistochemical and immunofluorescence analyses.

### Hind limb ischemia

7- to 8-week-old male C57Bl/6J mice (N=3) were anesthetized before undergoing unilateral proximal femoral artery ligation. The right femoral artery was gently isolated, ligated and excised distal to the deep femoral artery and 0.5 cm proximal to the bifurcation in saphenous and popliteal arteries, as previously described [7]. Contralateral legs underwent sham surgery. Seven days later, popliteal muscles from ischemic and non-ischemic hind limbs were harvested and processed for immunofluorescence analyses.

### Wound healing

The dorsum of anesthetized 7- to 8-week-old male C57Bl/6J mice (N=3) were shaved and a 6 mm-diameter full-thickness wound was performed on the dorsal midline using a biopsy punch. Two days later, mice were sacrificed and the skin was explanted and processed for immunofluorescence analysis. Skin hypoxic regions were detected by Hypoxyprobe-1 Plus Kit (Hypoxyprobe Inc.) according to the manufacturer's protocol. Mice were intraperitoneally injected with 60 mg/kg pimonidazole-HCl (25 mg/ml in 0.9% NaCl) 120 minutes before the sacrifice. Skin was collected and fixed in 4% PFA overnight, followed by washing in PBS, and then equilibrated in 30% sucrose and embedded in OCT for frozen sectioning. Hypoxyprobe was visualized by specific mouse IgG1 monoclonal antibody conjugated with FITC.

### Immunohistochemical analysis

10 μm-thick cryopreserved HCT-116 tumors sections (N=5 for each tumor) were fixed with PFA 4% and incubated overnight at 4°C with the following primary antibodies: rabbit-anti-human PlGF (1:250, Abcam), rabbit anti-Carbonic Anhydrase IX (1:1000, Novus Biologicals). The staining procedure was continued using specific secondary biotinylated antibody (all from DAKO) followed by the signal amplification performed using Vectastain elite ABC kit (Vector Laboratories). Diaminobenzidine tetrahydrochloride (Sigma) was used as a substrate for the visualization of antigen-antibody complex. Slides were counterstained with hematoxylin. Images were recorded with a digital camera Leica.

### Immunofluorescence analysis

10 μm-thick cryopreserved tumors, skin and muscles sections (N=5 sections for each sample) were fixed with PFA 4% and incubated overnight at 4°C with the following primary antibodies: rat anti-mouse PECAM-1 (anti-CD31; 1:200; BD Pharmingen), rabbit anti-LYVE-1 (1:100, Abcam), rat-anti-LYVE-1 (1:100, Novus Biologicals), goat anti-mouse PlGF-2 (1μg/ml, R&D Systems). Isotype IgG for anti-LYVE1 was substituted for the primary antibody to assess the specificity of the staining. Bound antibody was detected with Alexa fluor-conjugated secondary antibodies. Sections were mounted with Vectashield with DAPI (Vector Laboratories). Images were recorded with a digital camera Leica.

### Statistical analyses

Results are expressed as mean ± standard error of the mean (SEM), with P values < 0.05 considered statistically significant. Differences among groups were compared by 1-way ANOVA. Tukey HD test was used as a post hoc test to identify which group differences account for the significant overall ANOVA.

## Abbreviations

PlGF: Placental growth factor
LECs: lymphatic endothelial cells
VEGF: vascular endothelial growth factor
VEGFR1: VEGF receptor 1
HIF: hypoxia inducible factor
HRE: hypoxia responsive element
CAIX: carbonic anhydrase IX
LYVE1: lymphatic vessel endothelial hyaluronan receptor 1
IHC: immunohistochemistry
IF: immunofluorescence;
